# A comparison of the sealing abilities between Biodentine and MTA as root-end filling materials and their effects on bone healing in dogs after periradicular surgery

**DOI:** 10.1590/1678-7757-2018-0693

**Published:** 2019-10-07

**Authors:** Jing-jing Tang, Zong-shan Shen, Wei Qin, Zhengmei Lin

**Affiliations:** 1 Sun Yat-sen University, Guanghua School of Stomatology, Department of Operative Dentistry and Endodontics, Guangdong Provincial Key Laboratory of Stomatology, Guangzhou, China.; 2 Sun Yat-sen University, The Sixth Affiliated Hospital of Sun Yat-sen University, Department of Dentistry, Guangzhou, China.

**Keywords:** MTA, Biodentine, Periradicular surgery, Biocompatibility, Sealing ability

## Abstract

**Objectives:**

To compare the sealing ability and biocompatibility of Biodentine with mineral trioxide aggregate (MTA) when used as root-end filling materials.

**Methodology:**

The Cell Counting Kit-8 (CCK-8) assay was used to compare the cytotoxicity of MTA and Biodentine. Twenty-one extracted teeth with a single canal were immersed in an acidic silver nitrate solution after root-end filling. Then, the volume and depth of silver nitrate that infiltrated the apical portion of the teeth were analyzed using micro-computed tomography (micro-CT). Seventy-two roots from 3 female beagle dogs were randomly distributed into 3 groups and apical surgery was performed. After six months, the volume of the bone defect surrounding these roots was analyzed using micro-CT.

**Results:**

Based on the results of the CCK-8 assay, MTA and Biodentine did not show statistically significant differences in cytotoxicity (P>0.05). The volume and the depth of the infiltrated nitrate solution were greater in the MTA group than in the Biodentine group (P<0.05). The volume of the bone defect was larger in the MTA group than in the Biodentine group. However, the difference was not significant (P>0.05). The volumes of the bone defects in the MTA and Biodentine groups were smaller than the group without any filling materials (P<0.05).

**Conclusions:**

MTA and Biodentine exhibited comparable cellular biocompatibility. Biodentine showed a superior sealing ability to MTA in root-end filling. Both Biodentine and MTA promoted periradicular bone healing in beagle dog periradicular surgery models.

## Introduction

Root canal treatment is generally performed to treat dental pulp and periapical disease. When the root canal treatment and retreatment fail, periradicular surgery is the last hope for the affected teeth.^1^ The aim of periradicular surgery is not simply to remove infected apical tissue or the root tip, but most importantly is to reseal the root canal system.^2^ For this purpose, a root-end filling material must be applied to fill the root-end cavity and seal the exposed dentine during periradicular surgery. Hence, this material must possess sealing ability and biocompatibility. In addition, it should have antibacterial qualities and be easy to manipulate.^3^

Mineral trioxide aggregate (MTA), due to its superior characteristics compared to other traditional root-end filling materials such as Amalgam, gutta-percha and glass ionomer cement,^4^ is one of the most ideal root-end filling materials. It is also indicated in direct capping, apexification and perforation repair in dentistry. Its good biocompatibility and sealing ability have been verified in long-term clinical practice since the mid-1990s.^5^ However, the drawbacks of MTA, such as the potential for tooth discoloration, difficult handling characteristics and long setting time, limit its applications.^5^,^6^ Current research is actively seeking alternative materials to MTA.

Biodentine is a new tricalcium silicate-based cement material that was introduced in 2009. It has better handling properties and a shorter setting time than MTA. In addition, it possesses very similar physical properties to dentine and poses low risk of tooth discoloration. The powder mainly consists of tricalcium silicate, zirconium, and calcium carbonate. The liquid is composed of water, calcium chloride and hydrosoluble polymer.^7^

The Biodentine manufacturer claims that its applications are similar to MTA. For example, as a direct capping agent, Biodentine produces good results in asymptomatic vital permanent teeth with cariously exposed pulp.^8^–^11^ Moreover, it has also been applied in pulpotomy and apexification.^12^,^13^ Actually, the induction of mineralization of this bioactive material has been verified at the cell and molecular levels.^14^ As shown in the study by Daltoe, Biodentine induces similar levels of mineralization markers in the pulp compared with MTA. Together, animal models in which Biodentine has been employed in furcation perforation repair show that both Biodentine and MTA are excellent perforation repair materials, but MTA results in a greater frequency in complete sealing of the furcation perforation.^15^ However, the effect of Biodentine on the periapical tissue has not been completely elucidated. The apical circumstance is more complex than tooth furcation and root canals. Hence, the sealing ability and biocompatibility of Biodentine as a root-end filling material must be studied. This study aims to compare citocompatibility in human periodontal ligament cells and sealing ability of Biodentine to MTA when used as root-end filling materials and their effects on apical bone healing after periapical surgery in dogs.

## Methodology

### Cell Culture and Material Preparation

Three healthy, impacted third molars were extracted from three healthy male patients aged 18-22 years old. Informed consent was obtained from all patients before extraction. Briefly, the periodontal ligament was cut into pieces and digested in a solution of 2 mg/mL collagenase I (Invitrogen-Life Technologies, CA, USA) for 20 minutes at 37°C. The cells were centrifuged and resuspended. The cell suspension was seeded into a T-25 flask (Costar, Cambridge, USA) containing α-MEM supplemented with 20% FBS, 2% penicillin and streptomycin. Then, the cells were incubated in a 5% carbon dioxide incubator (Shellab, Cornelious, OR, USA) at 37°C. The cells were sub-cultured after reaching 80-85% confluence, and cells at the third passage were used on subsequent experiments. Periodontal ligament cells (PDLCs) were cultured in media supplemented with 10% FBS.

Both Biodentine (Septodont, Saint Maur des Fosses, France) and Pro-Root MTA (Dentsplay, Tulsa, USA) were prepared and set under aseptic conditions according to the manufacturer's instructions. After setting, 0.2 g of MTA or Biodentine was immersed in 1 mL of α-MEM supplemented with 10% FBS and incubated for 72 h at 37°C. Then, the materials were discarded and the eluate extracts were filtered with 0.22-µm pore size membranes (Millipore; Billerica, MA, USA).^16^,^17^

### Cell Counting Kit-8 (CCK-8) Assay

One hundred microliters of suspended PDLCs were dispensed in a 96-well plate (1000 cells/well). The cells had been pre-incubated in a 5% carbon dioxide incubator at 37°C for 24 hours. The cell culture media has been described previously and contained 100 μL of Biodentine and MTA in the appropriate wells. Periodontal cells treated with 3% hydrogen peroxide served as positive control and periodontal cells cultured with α-MEM supplemented with 10% FBS served as negative control. Then, the cells were incubated for 24, 48 or 72 hours. Next, 10 μL of the CCK-8 solution (Dojindo, Japan) were added to each well and incubated with the cells for 4 hours. Finally, the absorbance was measured at 450 nm. The relative growth rate (RGR) was calculated using the flowing formula:

### Micro-Computed Tomography Analysis of the Sealing Ability

Twenty-one extracted human teeth with a single canal were collected from the outpatient clinic of the Oral Surgery Department at Guanghua School of Stomatology, Sun Yat-sen University. The research was approved by the Ethics Committee of Guanghua School of Stomatology, Sun Yat-sen University (Approval Number ERC-2014-12). All teeth included in this study met the following criteria: a single root canal, mature apices, a lack of severe apical curvatures, and the absence of obvious fractures or cracks.

First, the crown was removed using a water-cooled diamond disc at a distance of 15 mm from the apex of the root. Then, the apical end was determined, and the standard crown-down technique was used to prepare the canal with a rotary ProTaper system (Dentsply Maillefer, Ballaigues, Switzerland). During instrumentation, irrigation was achieved with 5 mL of a 17% EDTA and 5.25% NaOCl solution (Sultan Healthcare Inc., Englewood, USA). After preparing the root canal, 5 mL of a 17% EDTA and 5.25% NaOCl solution were used to rinse the root canal. Finally, gutta-percha (Dentsply De Trey, Konstanz, Germany) and AH Plus (Dentsply De Trey, Konstanz, Germany) were used to complete the root obturation with the warm vertical compaction technique. The root orifices were filled with glass ion. Then, an area located 3 mm from the apex of all teeth was resected using a diamond burr. Root-ending cavities 3-mm deep and 1.0 mm in diameter were prepared using an ultrasonic tip (SATELEC, France).

The 21 teeth were divided into three groups: the Biodentine group (n = 7), the MTA group (n = 7), and the blank control group (n = 7). Teeth in the blank control group were not prepared or filled. Root-ending cavities were filled with Biodentine and MTA, accordingly. All teeth were incubated at 37°C for 7 days. Nail varnish was used to coat the teeth's apical surface to determine microleakage. All teeth were placed in a 50% silver nitrate solution (Guangzhou Chemical Reagent Factory, China) at room temperature for 12 hours.

The volume of the silver nitrate solution that infiltrated the root canal in all experimental groups was evaluated using micro-computed tomography (micro-CT) (Bruker micro-CT, Kontich, Belgium). Scanning parameters were 90 kV, 90 μA, and 708 ms with a voxel size of 18 μm. The scan data were obtained from all groups under the same conditions. Apical leakage was determined by measuring the amount of the silver nitrate solution that had infiltrated into the root canal. The data were analyzed using an image analysis software (VGStudio MAX 1.2.1, Heidelberg, Germany). The silver nitrate solution was recognized and then outlined according to the gray value of the image in each section. Working selections were reconstructed into 3-dimensional images using VGStudio MAX 1.2.1. The silver nitrate solution volumes and depth that infiltrated the root canal were calculated to assess the apical leakage in the MTA and Biodentine groups. All measurements were performed by two calibrated examiners in two different sessions. The interclass correlation coefficient (ICC) between two examiners was greater than 0.8. The ICC between different sessions by the same examiners was greater than 0.9.

Periradicular Surgery and Sample Analysis

This study was approved by the Animal Care and Use Committees of Sun Yat-sen University (IACUC-DB-15-1004). The first, second and third maxillary premolars and the first, second, third, and fourth mandibular permanent premolars of 3 female beagle dogs aged 18 months weighing an average of 10-11 kg were selected for the study (72 roots in total). The roots were randomly distributed into 3 groups: MTA (30 roots), Biodentine (30 roots), and negative control (12 roots) groups. Root-end cavities were not prepared or filled in the negative control group.

The animals were anesthetized via intramuscular injections of 3% pentobarbital (1 mL/kg). X-rays (60 kV, 7 mA, 0.2 s) were obtained to evaluate the canal system before the surgery. Root canal treatment was performed on each tooth. Briefly, when the experimental teeth were exposed, coronal access was established and the pulp tissue was removed. Initially, a 10# K file was used to reach the working length. Next, the ProTaper nickel-titanium system was used to prepare the root canal using an F4 file. During this procedure, saline and 3% hydrogen peroxide were used to rinse the root canal. The entire canal was filled with gutta-percha supplemented with AH Plus, and the root orifices were sealed with glass ion. X-rays were used to assess the root canal filling.

The procedures used for periradicular surgery were performed according to the method described by Apaydin.^18^ Briefly, a full-thickness mucoperiosteal buccal flap with two releasing incisions was created. The bone from the buccal side was removed with a round bar, and the bone defects were shaped with a high-speed handpiece with saline irrigation; the procedure stopped when the diameter of the defects reached 4 mm. The root ends were resected in all groups with a fissure burr approximately 3 mm from the apex. A root-ending cavity with a depth of 3 mm was created with an ultrasonic unit and filled with MTA or Biodentine according to the manufacturer's recommendations. The mucoperiosteal flaps were sutured with 4-0 silk sutures. After surgery, the animals were placed on a soft diet. After six months, the animals were euthanized without pain.

All experimental roots were dissected and scanned using micro-CT. Scanning parameters were 70 kV, 200 μA, and 300 ms with a voxel size of 34.4 μm. The data were analyzed using VGStudio MAX 1.2.1 software. The bone defects were recognized and labeled according to the gray value of the image of each section. Working selections were reconstructed into 3-dimensional images using VGStudio MAX 1.2.1. Bone defect volumes were calculated to assess the apical healing in the MTA and Biodentine groups. All measurements were performed by two calibrated examiners in two different sessions.

### Statistical Analyses

Statistical analyses were performed using one-way analysis of variance followed by the Student-Newman-Keuls test with SPSS 13.0 software. 95% confidence intervals were determined. A *P*-value less than 0.05 was considered statistically significant.

## Results

### Toxicity of the Materials

**Figure 1:**
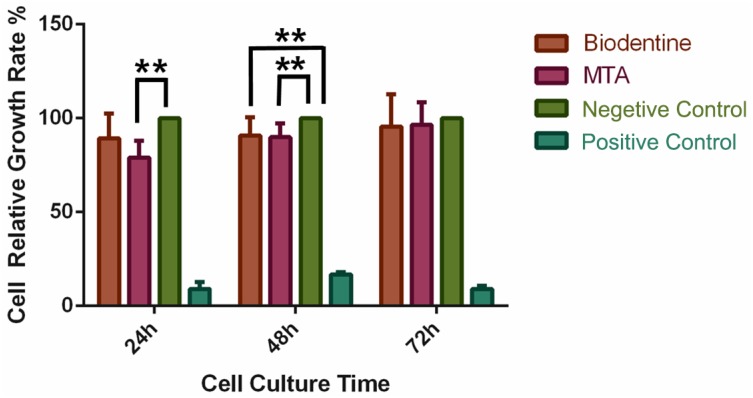
Effects of MTA and BD on human periodontal ligament cells (hPDLCs). hPDLCs were cultured with α-MEM containing MTA or BD in a 96-well plate for 24, 48, or 72 hours. The relative growth rate (RGR) was evaluated using a CCK-8 assay. The RGR of the negative control (NC) group was set to 100%, and other groups were compared to the NC group. **P < 0.01.

The RGR was set to 100% in the NC group. At 24 hours, a lower RGR was observed for PDLCs in the MTA group than in the negative control group (NC) (*P*<0.01), whereas no significant difference was observed between the Biodentine group and the NC group (*P*>0.05). After 48 hours, the RGR of PDLCs in both the MTA and Biodentine groups was lower than the NC group (*P*<0.01), but no significant differences were observed between the two experimental groups (*P*>0.05). After 72 hours, no significant differences were observed among the MTA, Biodentine and NC groups (Figure 1).

### Determination of Microleakage Using a Micro-CT Analysis

**Figure 2:**
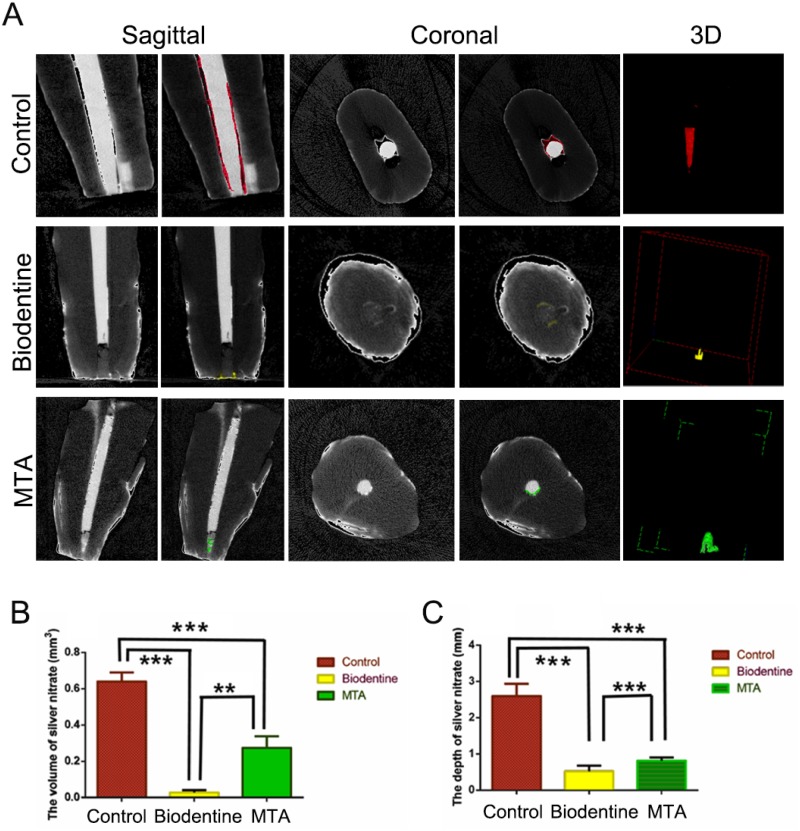
Determination of microleakage using a micro-CT analysis. (A) Micro-CT images showing the volume of the silver nitrate solution that infiltrated the root canal in each experimental group. (B) Quantitative analysis of the volume of the nitrate solution that infiltrated the root canal. (C) Quantitative analysis of the depth of the nitrate solution that infiltrated the root canal. **P < 0.01 and ***P < 0.001.

Based on the quantitative analysis (Figure 2A), a larger volume of the infiltrated nitrate solution was observed in the MTA group than in the Biodentine group (Figure 2B, *P*=0.014). The infiltration depth was also greater in the MTA group than in the Biodentine group (Figure 2C, *P*=0.005). Both groups showed less apical microleakage than the control group, according to both depth and volume (Figure 2C, *P*<0.001).

### Evaluation of Bone Formation Using Micro-CT

**Figure 3:**
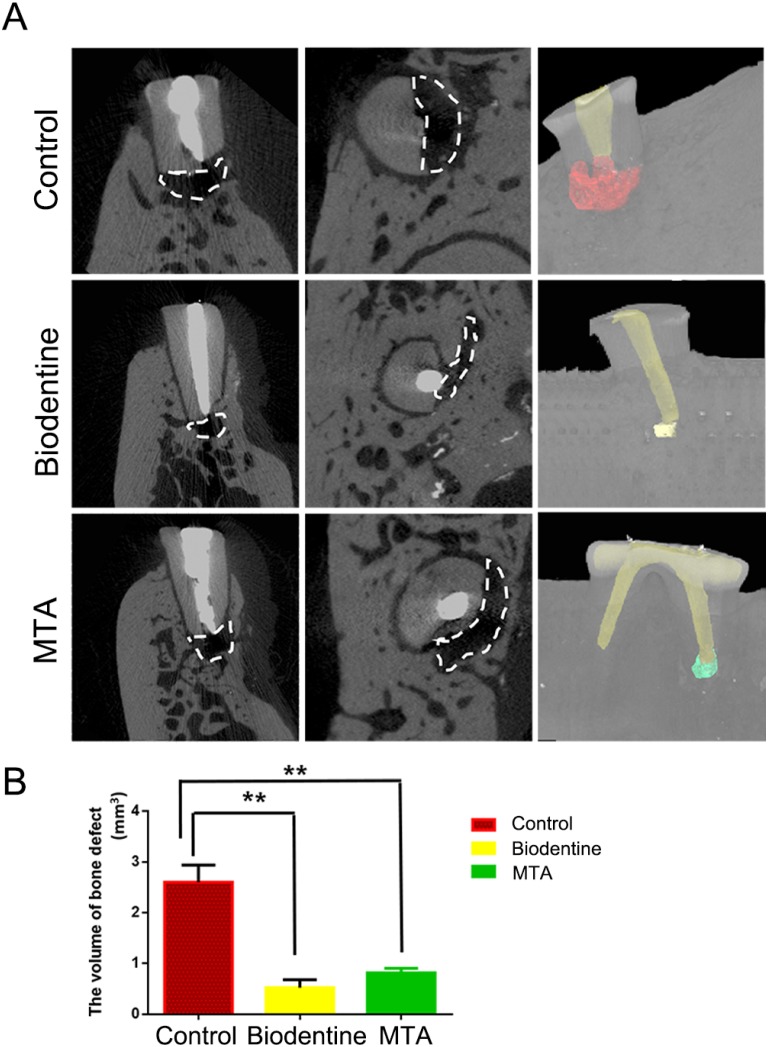
Evaluation of bone formation using micro-CT after six months of root-end filling of dogs' teeth. (A) Volume of bone defects in the negative control group, BD root-end filling and MTA root-end filling groups. (B) Quantitative analysis of bone defect volumes in each group. **P < 0.01.

The volume of the bone defect in the MTA group was larger than in the Biodentine group. However, the difference observed between the MTA and Biodentine groups was not significant (*P*>0.05). The volumes of the bone defects in the MTA and Biodentine groups were both smaller than the defects in the NC group (*P*<0.05). The statistical evaluation of tissue formation at six months after surgery indicated that both MTA and Biodentine promoted bone tissue regeneration (Figure 3).

## Discussion

In the present study, Biodentine possessed a similar sealing ability and biocompatibility to MTA. Thus, Biodentine has great potential as an alternative to MTA in periradicular surgery.

According to the results of the CCK-8 assay, both MTA and Biodentine exhibited low levels of cytotoxicity at 48 hours, but not at 72 hours. The low levels of cytotoxicity induced by both materials in the early stage can be due to a hydration reaction. MTA and Biodentine contain calcium silicate, so calcium hydroxide would be created in a hydration reaction when calcium silicate reacts with water. Calcium hydroxide can increase the pH of the culture media, which damages PDSCs. Other biocompatibility studies showed that MTA and Biodentine exhibited slight cytotoxicity, but the toxic effects were less than in other materials, such as octacalcium phosphate and IRM.^19^–^21^

Sealing ability is a key characteristic of root-end materials that determines the success of periapical surgery. The common methodologies used to assess apical and coronal leakage include the dye penetration and extraction method, the fluid filtration or transportation method, and the bacteria and toxin infiltration method.^22^ Due to its low cost and simplicity, the dye penetration and extraction method has been the technique preferred by most researchers. However, the teeth must be sectioned longitudinally or transversely to examine the extent of dye penetration. With this technique, the sample is destroyed and only the sectioned planes are evaluated.^23^ In previous studies, researchers obtained three 1-mm thick transverse sections by sectioning the apical portions of the roots, and then used confocal laser scanning microscopy (CLSM) to determine gaps between the root-end filling materials and the dentin.^24^ In these experiments, the results only reflected the two-dimensional gaps. In addition, because transverse sections and the selected areas measured with CLSM are limited, the results were inevitably affected by subjective factors.

Interestingly, a study by Kakaboura, et al.^25^ (2007) evaluated the 3D-marginal adaptation between dentine and resin composites using computerized X-ray microtomography. This method possesses unique advantages such as full 3D-fidelity, enabling a quantitative evaluation of the interfacial adaptation at any site and direction.^26^ We evaluated the microleakage between dentine and root-end filling materials with a similar method described by Kakaboura, et al.^25^ (2007). The entire leakage volume and depth were imaged, with no destruction of the samples. In the present study, a greater volume and depth of the infiltrated nitrate solution was observed in the MTA group than in the Biodentine group. However, according to Soundappan, et al.^27^ (2014), the marginal adaptation of MTA and IRM was superior to Biodentine when used as root-end filling materials. These divergent results may be attributed to the differences in the accuracy of these methods. We measured microleakage with 3D imaging, which was more accurate than a simple qualitative evaluation of microleakage in transverse sections. Based on our 3D evaluation, we conclude that the sealing ability of Biodentine is adequate when applied in root-end fillings. The limitation of this study is that we only scanned the teeth after immersion. The results might be more convincing if we obtain the scan data before immersion in silver nitrate solution and compare them with the scanned data after immersion.

Furthermore, we explored the biological effect of Biodentine *in vivo*. Although the beagle dog is more expensive and difficult to operate upon, it is still more suitable than other animals, such as mice and rats, as a model of the apical responses in humans. In addition, the teeth of other small animals are too small to operate on using the root-end filling technique. Hence, we chose beagle dogs as a periradicular surgery model. The design of the animal experiment used in the present study differs from other similar studies because apical periodontitis was not induced before treatment. The aim of this design was to eliminate the confounding effect of induced periodontitis on assessing the biocompatibility of Biodentine. For instance, when treatment failure occurs in a tooth with induced periodontitis, a determination of whether the cause of failure was attributed to the material would be difficult.^28^

The beagle dog model has been widely used to evaluate the characteristics of dental materials, including Biodentine. For example, Silva, et al.^15^ (2017). showed that Biodentine can be used as a furcation perforation repair material in the beagle dog model, with comparable results to MTA. According to De Rossi et al.^29^ (2014), Biodentine and MTA both facilitate mineralized tissue bridge formation after pulpotomy. However, no study has compared Biodentine and MTA when applied as root-end filling materials *in vivo*. In the present beagle dog periradicular surgery model, micro-CT was used to evaluate the bone healing process and the results showed that both materials exhibited good effects on periradicular bone healing. As shown in the study by Daltoe, et al.^14^ (2016), the ability of Biodentine to upregulate mineralization markers was similar to MTA. In addition, Ho, et al.^30^ (2018) confirmed the potential utility of Biodentine in dental and bone regeneration using three-dimensional printed Biodentine/polycaprolactone composite scaffolds. These studies have confirmed that Biodentine promotes bone healing.

## Conclusions

Biodentine and MTA showed comparable cell biocompatibility. Biodentine was superior to MTA in terms of sealing ability when used as a root-end filling material. Both Biodentine and MTA promoted periradicular bone healing after periradicular surgery in beagle dog models.
